# JAXA protein crystallization in space: ongoing improvements for growing high-quality crystals

**DOI:** 10.1107/S0909049513021596

**Published:** 2013-09-26

**Authors:** Sachiko Takahashi, Kazunori Ohta, Naoki Furubayashi, Bin Yan, Misako Koga, Yoshio Wada, Mitsugu Yamada, Koji Inaka, Hiroaki Tanaka, Hiroshi Miyoshi, Tomoyuki Kobayashi, Shigeki Kamigaichi

**Affiliations:** a Confocal Science Inc., Hayakawa 2nd Building 7F, 2-12-2 Iwamoto-cho, Chiyoda-ku, Tokyo 101-0032, Japan; b Japan Aerospace Exploration Agency, 2-1-1 Sengen, Tsukuba, Ibaraki 305-8505, Japan; c Maruwa Foods and Biosciences Inc., 170-1 Tsutsui-cho, Yamatokoriyama, Nara 639-1123, Japan

**Keywords:** microgravity, protein crystal, JAXA, counter-diffusion, Japan Experiment Module ‘Kibo’, protein depletion zone, impurity depletion zone

## Abstract

The Japan Aerospace Exploration Agency (JAXA) started a high-quality protein crystal growth project, now called JAXA PCG, on the International Space Station (ISS) in 2002. Using the counter-diffusion technique, 14 sessions of experiments have been performed as of 2012 with 580 proteins crystallized in total. Over the course of these experiments, a user-friendly interface framework for high accessibility has been constructed and crystallization techniques improved; devices to maximize the use of the microgravity environment have been designed, resulting in some high-resolution crystal growth. If crystallization conditions were carefully fixed in ground-based experiments, high-quality protein crystals grew in microgravity in many experiments on the ISS, especially when a highly homogeneous protein sample and a viscous crystallization solution were employed. In this article, the current status of JAXA PCG is discussed, and a rational approach to high-quality protein crystal growth in microgravity based on numerical analyses is explained.

## Abstract 2

The Japan Aerospace Exploration Agency’s ‘high-quality protein crystal growth’ project is introduced. If crystallization conditions were carefully fixed in ground-based experiments, high-quality protein crystals grew in microgravity in many experiments on the International Space Station, especially when a highly homogeneous protein sample and a viscous crystallization solution were employed.

## 1. Introduction

With regard to the X-ray diffraction analysis of protein crystals, the technique has been progressing over the last decades, from protein sample preparations and optimization of crystallization conditions to improvements in hardware and software for X-ray diffraction experiments and beyond. For X-ray crystallography, growing high-quality crystals is still the biggest hurdle (McPherson, 1999 ▶).

Since the 1980s, many crystallization experiments in microgravity have been performed in the hope of finding a solution to this problem, because microgravity may encourage good crystal growth by minimizing convective flow (McPherson, 1999 ▶). Littke & John (1986 ▶) successfully obtained large lysozyme crystals in space in 1983. Since then, many crystallization trials have been performed in microgravity (Snell & Helliwell, 2005 ▶; Vergara *et al.*, 2005 ▶). However, in 2000, the National Research Council of the USA reported that the impact of microgravity crystallization on structural biology as a whole is extremely limited (http://www.nap.edu/books/0309069750/html) before fully understanding the differences in quality between microgravity- and ground-grown crystals. The many parameters that must be considered to judge whether space-grown crystals are superior to ground-grown ones make this a complicated analysis.

On the other hand, Snell & Helliwell (2005 ▶) noted that the success rate of space experiments based on the criteria of improved diffraction resolution increased to 60% when the analysis was limited to proteins that have flown four or more times. This implies that microgravity would provide an advantage in obtaining better-quality crystals if the crystallization technique was well optimized. The positive effects of microgravity on crystal growth, such as the formation of a protein depletion zone (PDZ) (Otálora *et al.*, 2001 ▶) and impurity depletion zone (IDZ) (Thomas *et al.*, 2000 ▶; Chernov *et al.*, 2001 ▶) around a growing crystal, were reported by researchers of crystal growth mechanisms. These depletion zones are considered to cause lower concentrations of protein and impurities on the surface of a crystal, resulting in high-quality crystal growth in microgravity.

**Figure 1:**
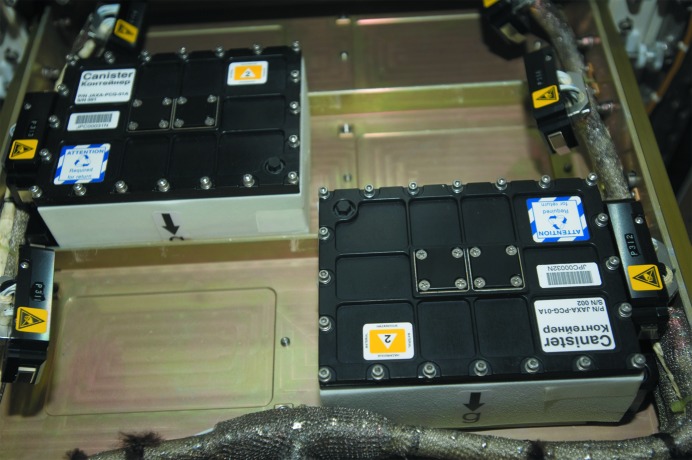
Protein crystallization device in JEM (by courtesy of JAXA/NASA). Two canisters are placed in the PCRF in JEM which can maintain a temperature of 293 K.

The Japan Aerospace Exploration Agency (JAXA) has conducted a series of high-quality protein crystal growth experiments [JAXA-GCF (Sato *et al.*, 2006 ▶), as a part of its space utilization program, JAXA-NGCF, JAXA PCG in chronological order] on the ISS since 2002 following the pioneering work of the Granada Crystallization Facility (García-Ruiz *et al.*, 2002 ▶; Otálora *et al.*, 2009 ▶). JAXA has aimed to regularly obtain high-quality crystals of useful proteins, so that microgravity crystallization can become one of the choices for researchers to consider in their experiment designs. For this purpose, a standard user-friendly experiment protocol was established to expedite the process from the acceptance of the samples to the launch. Also, numerical analyses were used to help establish a rational approach to increasing the probability of high-quality crystal growth through the enhancement of the PDZ and IDZ (Tanaka *et al.*, 2004*a* ▶, 2012 ▶; Inaka *et al.*, 2012 ▶). For transportation to and from the ISS, Russian Progress cargo ships and the Soyuz spacecraft have been used. Since 2008, these crystallization experiments have been performed in the Protein Crystallization Research Facility (PCRF) on board the Japanese Experiment Module ‘Kibo’ (JEM) (Fig. 1 ▶). Russian and Malaysian researchers have been participating in the experiments. Technical achievements in high-quality crystal growth and upgrades in the support system for users continue to advance, and, at present, if crystallization conditions are properly optimized, the reproducibility of high-quality crystal growth is a likely expectation.

## 2. Rational approach for space experiments

PDZ and IDZ are considered to be formed around a growing crystal through the depression of the convective flow in microgravity (Otálora *et al.*, 2001 ▶; Thomas *et al.*, 2000 ▶; Chernov *et al.*, 2001 ▶). This can be explained using a simplified spherical model under steady conditions. The effects of PDZ can be expressed as the driving force ratio (DFR) and the effects of IDZ as the impurity ratio (IR) as described previously (Tanaka *et al.*, 2004*a* ▶, 2012 ▶). These ratios can be expressed as
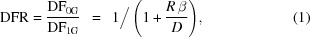


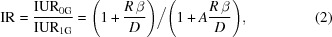
where 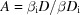, *R* is the radius of a crystal, β and β_i_ are kinetic coefficients for the protein and the impurity molecules, and *D* and *D*_i_ are diffusion coefficients of the protein and the impurity molecules. From these equations it was concluded that DFR and IR are lower if *R*β/*D* is larger. This means that PDZ and IDZ become more significant when a crystal grows larger (*R* is larger), the uptake of the molecules into the crystal is faster (β is larger), and the diffusion of the molecules is slower (*D* is smaller). As suggested in our previous report (Inaka *et al.*, 2012 ▶), if the usual size of the crystal in an X-ray diffraction experiment is around 0.1 mm on a side, then IR, not DFR, is significantly decreased. This implies that it is the IDZ that mainly affects high-quality crystal growth in microgravity.

We introduced an equation to roughly estimate *D* (Tanaka *et al.*, 2006 ▶) and used the experimental method to estimate β (Tanaka *et al.*, 2004*a* ▶). Using *D* and β, we can estimate whether microgravity will positively affect each crystallization condition before performing a space experiment, and, if necessary, we may change a crystallization condition to one more suited to microgravity experiments. We calculated that if *D*/β < 3 mm, better quality crystals were grown in microgravity for about 70% of the proteins (Table 1 ▶). In Table 1 ▶, the effective sample means that the crystal quality was improved, indexed not only by the maximum resolution or the mosaicity of the crystal but also by the improvement of the crystal shape from a cluster-like morphology to a fine single crystal. For the remaining 30% of the proteins, crystals of improved quality could not be grown even when *D*/β was less than 3 mm. The major causes for this were some differences in the sample lot used for the optimization of crystallization conditions and the one used in the space experiment. In some other cases, some deterioration of the space-grown crystals was caused by temperature surging up to almost 303 K during the retrieval flight from the ISS.

To lower *D*/β for maximizing the effects of microgravity on crystal growth, the first approach is to lower *D*, which can be achieved by preparing a high-viscosity crystallization solution using such additives as high-molecular-weight polyethylene glycol (PEG). In a previous report (Yamanaka *et al.*, 2011 ▶), we found that the salt concentration in a PEG solution is crucial and that it is possible to estimate the lowest effective salt concentration. Therefore, PEG became more widely used in crystallization solutions for our experiments, even when the crystallization conditions did not originally contain PEG, although there are still certain proteins which will not crystallize with PEG. As a result we have obtained some good results with such proteins as lipocalin-type prostaglandin D synthase (L-PGDS) (Inaka *et al.*, 2011 ▶) (data collection up to 1.06 Å for space-grown crystals and 1.30 Å for ground-grown crystals) and alpha-amylase (visual observation up to 0.85 Å and data collection up to 1.0 Å for space-grown crystals and visual observation up to 1.0 Å and data collection up to 1.1 Å for ground-grown crystals). Using PEG in the crystallization solution has dramatically increased the quality of crystals grown in microgravity in these cases.

Further purification of protein samples is effective in increasing β. We applied high-performance ion-exchange column chromatography to a lysozyme sample to make it electrically homogeneous, and found out that it made β several-fold larger (Tanaka *et al.*, 2012 ▶). We speculated that the impurities (sometimes the same protein molecule with a different electric charge) which were separated out by ion-exchange column chromatography might affect the crystal growth and decrease β. Actually, we applied this method to some other proteins, including alpha-amylase, hematopoietic prostaglandin D synthase (H-PGDS) (Takahashi *et al.*, 2010 ▶; Tanaka *et al.*, 2011 ▶) and L-PGDS (Inaka *et al.*, 2011 ▶), and obtained better results. Alpha-amylase has been crystallized in almost every JAXA PCG experiment as a benchmark protein and we have always obtained high-quality crystals from which X-ray diffraction was visually observed around 1.0 Å with improved morphologies. H-PGDS has been crystallized in space several times with various ligands since; for some cases, we have successfully obtained a data set of X-ray diffraction of almost 1.0 Å resolution (Tanaka *et al.*, 2011 ▶). L-PGDS has been crystallized several times in space and has always yielded high-quality crystals by which we can obtain data sets of X-ray diffraction at around 1.0 Å resolution (Inaka *et al.*, 2011 ▶). Therefore, we concluded that if crystallization conditions are properly optimized, we can obtain good-quality crystals in space, and we can reproduce these results.

## 3. Standard protocol of JAXA PCG

**Figure 2:**
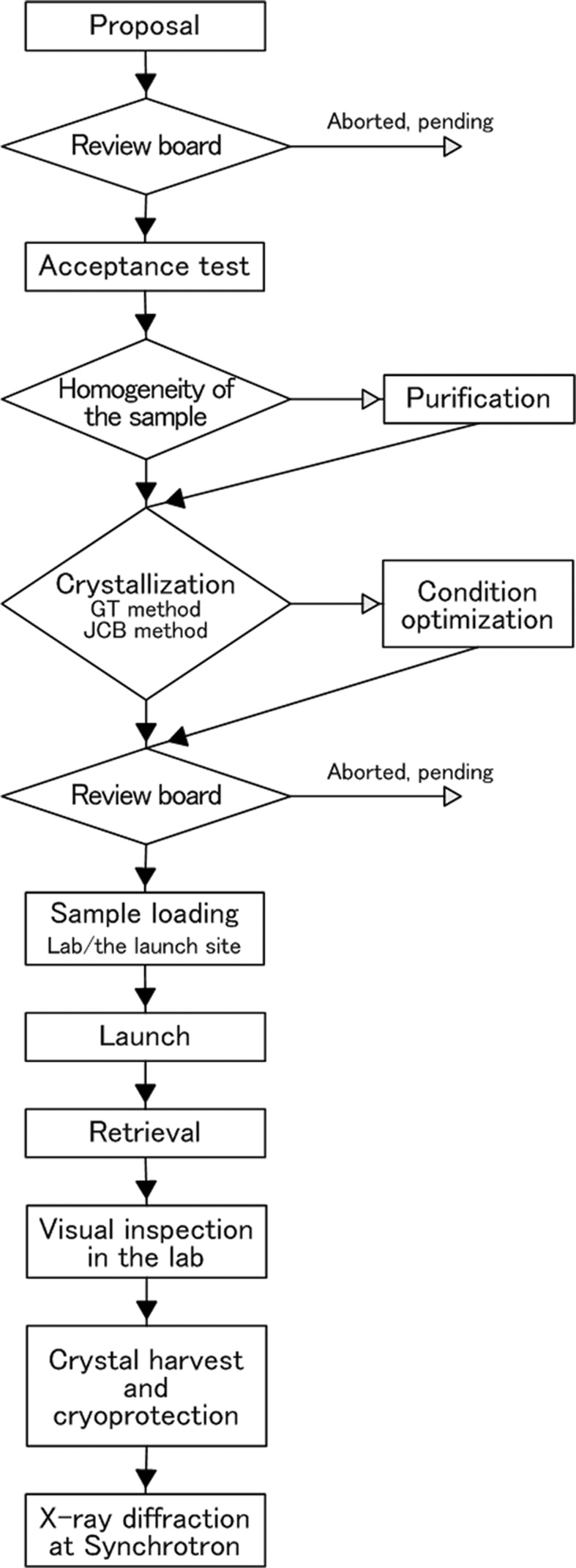
Standard protocol for crystallization experiments of JAXA PCG.

Based on the rational approach for space experiments, JAXA set a goal for high-quality protein crystal growth in microgravity and designed a standard protocol for the efficient performance of space experiments as shown in Fig. 2 ▶.

The protocol initiates with a proposal submitted by potential users. Based on the datasheet of protein information and crystallization history in a laboratory, the review board decides whether or not to accept the proposal. At present, only a protein which is stable at 293 K for more than several months is accepted since the PCRF is kept at this temperature.

As aforementioned, sample homogeneity is crucial for space experiments. Therefore, we perform an acceptance test to check this with SDS-PAGE, native-PAGE, dynamic light scattering, high-performance liquid chromatography, and a crystallization check using the vapor-diffusion method. The last check is performed under the same conditions as the potential users’ crystallizations since most of the samples are crystallized in the laboratory by the vapor-diffusion method. We found that heterogeneity of the sample was often observed even though the crystal grew well. In such a case, we not only ask users to improve the sample preparation but also apply a further sample purification step using high-performance ion-exchange column chromatography in the hope of increasing β. This purification step often makes crystal growth reproducible sometimes with a better and larger shape.

After the acceptance test, we convert the crystallization conditions from vapor-diffusion to counter-diffusion (García-Ruiz & Moreno, 1994 ▶; García-Ruiz *et al.*, 2002 ▶; García-Ruiz, 2003 ▶), sometimes using a one-dimensional simulation program developed by JAXA. We fine-tune the conditions using two steps of the counter-diffusion method. The first is the gel-tube method (Tanaka *et al.*, 2004*b* ▶) and the second is the JAXA Crystallization Box (JCB) method which is identical to the flight configuration. When we have to optimize a lot of crystallization conditions, the gel-tube method is most suitable because it is inexpensive and easy to assemble. The counter-diffusion method differs from the vapor-diffusion method on such points as the optimum concentration of protein and precipitant, and the nucleation possibility, *etc*. When the possibility of nucleation is very low in the counter-diffusion method, we apply a micro-seeding technique. Only samples which produce crystal growth with the counter-diffusion method are considered ready for launch by the review board.

For launch, all samples are loaded by JAXA in the laboratories in Japan for Japanese and Malaysian users and in Moscow for Russian users or at the launch site in Baikonur, Kazakhstan, depending on the profile of crystal growth. If performed in Japan, sample loading takes place about two weeks before launch. Samples which start nucleation earlier are loaded in Baikonur. The duration of crystallization in space ranges from six weeks to four months. The ground-control experiment is always performed using the same protein sample and the same crystallization device as the space experiment.

After the retrieval of the crystals from space, they are quickly inspected by microscope in Japan before being handed to users. We rate the appearance of the crystals to present information to the users and for JAXA’s data accumulation. If a crystal obtains a high score and the user requires assistance, JAXA offers support for harvesting crystals from the capillary and the cryo-protection of the crystals.

As described above, the JAXA PCG standard protocol is a total support system. In order to obtain meaningful results, we believe it is crucial that researchers using space for crystallization experiments are taken care of from the beginning of their experiment to the end. This is because opportunities to grow crystals in space are both infrequent and different from ordinary crystallization in the laboratory. Only a few users have the experience and know-how to obtain the best results on their own.

## 4. Crystallization device in the Japanese experiment module Kibo

From the beginning of JAXA-GCF, we adopted the Granada Crystallization Box (GCB) as a crystallization device using the counter-diffusion method (García-Ruiz & Moreno, 1994 ▶; García-Ruiz *et al.*, 2002 ▶; García-Ruiz, 2003 ▶). Since the sixth flight of JAXA-GCF in 2005, we modified the GCB to make the JCB the main crystallization device. The main features of the JCB are the separate crystallization cells and the volumetric efficiency. Capillaries containing protein solutions are plugged with agarose gel and inserted into tubes containing crystallization solutions. Using the JCB, we can apply up to 144 different crystallization conditions in a limited space (about 1 l; 100 mm × 150 mm × 65 mm). The assembly of the JCB has been described previously (Takahashi *et al.*, 2010 ▶). As shown in Figs. 1 ▶ and 3 ▶, two JCBs are coupled in a tray, and four trays are stacked and fastened with Teflon tape (JCB unit). Then the JCB unit is wrapped in an inner thermo-sealed sealing bag (Star plastic, Osaka, Japan), placed in an outer sealing bag (Mitsubishi Plastic, Tokyo, Japan), and then a metallic holder. Then three holders are loaded into a canister which can be connected to the PCRF on the JEM for temperature control (293 K).

During transport to and from the ISS, the temperature control is completely passive. Sometimes the temperature inside the Soyuz rises above 303 K. Therefore, a phase transition material, heptadecane, in a number of plastic bags and a vacuum insulator are positioned to cover the canister. Since the melting point of heptadecane is 295 K, it can keep the protein samples at lower than 295 K until it melts completely. If we use enough of the material, the temperature stays stable.

## 5. Current status of JAXA PCG

The usefulness of space experiments is shown in Table 2 ▶ at each step of the standard protocol in Fig. 2 ▶. In the table, a ‘good result’ means that the quality of the crystal grown in space, indexed by maximum resolution, mosaicity or improved morphology, was higher than that of the best ground-grown crystal which was grown with the usual vapor-diffusion method or the counter-diffusion method as a ground-control experiment, and the X-ray diffraction data set advanced the research of the users. Among the samples whose crystallization conditions were well optimized, it was found that good results were obtained in 50% of the launched samples when the acceptance test result was good; in 37% of the launched samples when the further purification step was applied; and only in 5% of the launched samples when further purification was not applied though the acceptance test result was less than optimum. Therefore, we concluded that it is very important to improve the quality of the protein sample for successful space experiments.

## 6. Upgrade of crystallization cell

We are now preparing an upgrade of the JCB, the JCB-SGT. The crystallization cell is made of Techbarrier (Mitsubishi Plastics, Tokyo, Japan) so no water vapor or oxygen can permeate it. It can contain about 3.5 times as much reservoir solution as the JCB with the same volumetric efficiency, so the precipitant will not be diluted in the capillary and its concentration in the protein solution can be higher than that in the JCB. Moreover, the assembly of the JCB-SGT is much easier than the JCB, reducing the time for sample loading.

In 2008 and 2010, we prepared crystallization cells of 7.2 mm diameter to grow large crystals with the intention of using them in neutron diffraction experiments and successfully obtained an alpha-amylase crystal of 5 mm × 2  mm× 2 mm, lysozyme crystal of 1.2 mm × 1.2 mm × 1.2 mm, and glucose isomerase crystal of 1 mm × 1 mm × 1 mm in space. We are now in the development phase of an upgraded version of crystallization cells for large crystals (JCB-SLC).

## 7. Conclusion

JAXA has developed technologies for the purpose of maximizing the usage of the microgravity environment. Our aim for the future is to make crystallization experiments in space a customary platform for researchers. In the 1990s, it was said that the contribution of space crystallization to structural biology was only limited. As of now, however, in JAXA PCG, in about 70% of the cases, we have obtained crystals of improved quality in space when the crystallization conditions were properly optimized. We will distribute our methodology for space crystallization to users who want to obtain good-quality crystals. From our almost ten years of continuous experience in JAXA PCG, we know that crystallization in space can generate very high quality protein crystals if the protein sample is homogeneous and the crystallization conditions are finely tuned. The most integral part of successful crystal growth in space is to make sure that high-quality crystals are able to grow under the same conditions on the ground beforehand. With these things in place, space-grown crystals can yield superior X-ray diffraction data which is very unlikely with crystals grown in a laboratory. As we obtain good results from space-grown crystals, space experiments to test results using different ligands may contribute to obtaining high-resolution data of proteins for drug design *etc*.

There are still more details to be elucidated, but when our technique is more sophisticated and can be applied to a larger variety of protein samples, crystallization in microgravity will be a more useful methodology and will improve the efficiency of the structural analysis of proteins.

## Figures and Tables

**Figure 3:**
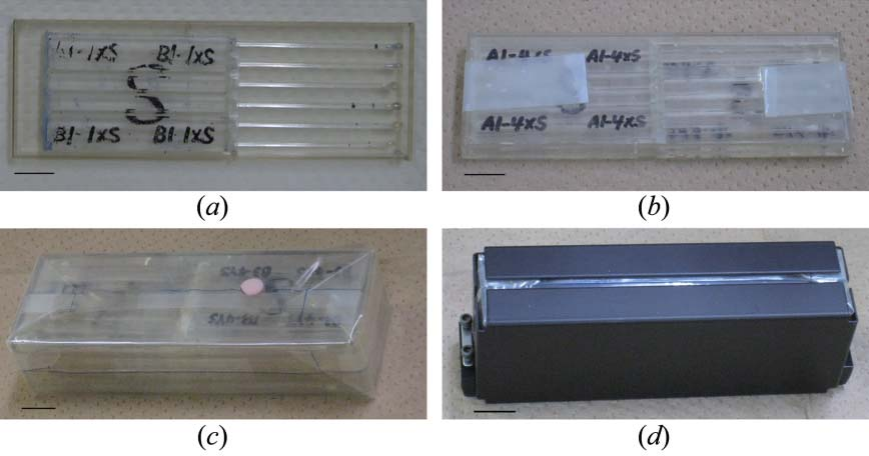
Assembly of the crystallization device for launch. (*a*) The JCB consists of six capillaries in a syringe case. Capillaries containing protein solutions are plugged with agarose gel and inserted into tubes, or cells, containing crystallization solutions. (*b*) To save space, two JCBs are coupled, *i.e.* the capillaries of two JCBs are positioned to fit between the slots of each other and they are put in a tray. (*c*) Four trays are stacked creating a JCB unit and wrapped in an inner bag and an outer bag. (*d*) A metallic holder contains one JCB unit. The scale bars correspond to 10 mm.

**Table 1:** Usefulness of the space experiment indexed by *D*/β The rates of microgravity experiments successfully obtaining higher-quality crystals, judged by higher maximum X-ray resolution, lower mosaicity and/or better morphology, in accordance with the *D*/β value, are shown. The data were collected from the protein crystals obtained from flight JAXA PCG#1 to flight JAXA PCG#5. Only the crystals for which the *D*/β value could be calculated were used.

	*D*/β			
	<1.0	1.0–3.0	3.0–10.0	>10.0
Number of samples	10	13	14	27
Number of effective samples	7/10 (70%)	9/13 (69%)	7/14 (50%)	11/27 (41%)
Average crystal radius (mm)	0.089	0.117	0.087	0.059

**Table 2:** Usefulness of space experiments at each step of the standard protocol The usefulness of space experiments is calculated at each step of the standard protocol. Among the samples whose crystallization conditions were well optimized, it was found that ‘good results’ were obtained in 50% of the launched samples when the acceptance test result was good; in 37% of the launched samples when the further purification step was applied; and only in 5% of the launched samples when the further purification was not applied though the acceptance test result was less than optimum. A ‘good result’ means that the quality of the crystals grown in space, indexed by maximum resolution or mosaicity, was higher than that of the best ground-grown crystal, and the X-ray diffraction data set advanced the research of the users.

Project	JAXA PCG#1~#5		
Number of proteins	215		
Acceptance test	Good	Less than optinum	
	61/215 (28%)	154/215 (72%)	
Further purification	N/A	Applied	Not applied
		64/154 (42%)	90/154 (58%)
Optimization and launch	44/61 (72%)	41/64 (64%)	59/90 (66%)
Single crystal	33/44 (75%)	29/41 (71%)	29/59 (49%)
Good result	22/44 (50%)	15/41 (37%)	3/59 (5%)
Success rate	40/215 (19%)		
